# Evaluating the Effectiveness of the Annual Physical Training Plan for Masters +45 Women Half Marathon Athletes: A Guideline Model for Good Practices for Programming Effort Volume and Intensity

**DOI:** 10.3390/sports12090256

**Published:** 2024-09-14

**Authors:** Daniela Baba, Raluca Mijaica, Florentina Nechita, Lorand Balint

**Affiliations:** 1 Sports Science and Physical Education, IOSUD—Transilvania University of Brasov, 500036 Brasov, Romania; daniela.baba@unitbv.ro; 2 Department of Physical Education and Special Motricity, Faculty of Physical Education and Mountain Sports, Transilvania University of Brasov, 500036 Brasov, Romania; florentina.nechita@unitbv.ro (F.N.); lbalint@unitbv.ro (L.B.)

**Keywords:** annual physical training program, half marathon, master +45, women, training volume planning, training intensity, athletic performance

## Abstract

This study presents the implementation and results of the Annual Physical Training Program for Masters +45 Half Marathoners (PASm-12), focused on optimizing athletic performance through rigorous planning of training volume and intensity. PASm-12, structured over 12 mesocycles and 52 microcycles, was applied to 6 female runners with over 10 years of experience. The results indicate that the total running volume achieved (2347 km) was 90.2% of the volume proposed by PASm-12 (2603.2 km), with statistically significant differences in most mesocycles (Cohen’s f^2^ = 6.24, t = −5.997, *p* = 0.002, indicating a large effect size). The training intensity was achieved at an average of 94.8% of what was proposed by PASm-12, with significant differences in several mesocycles (Cohen’s f^2^ = 0.45, t = −1.972 to −3.984, *p* < 0.05, indicating a moderate to large effect size). The female runners’ performances in field tests generally showed faster times than the maximum and average values proposed in PASm-12, with the exception of the final competition, where performance was slightly lower due to external competitive factors (Cohen’s d = −0.53, t = −1.192, *p* = 0.3). This plan, demonstrating good practice, could serve as a guideline model for amateur runners who do not have specialist counseling. PASm-12 can reduce the risk of injury, prevent excessive fatigue, and support ongoing participation in sports activities. Additionally, the implementation of this plan could provide amateur runners with a safe and effective training structure, contributing to improved health and athletic performance.

## 1. Introduction

A half marathon is an athletic event involving a 21.0975 km race, half the distance of a marathon [1]. In sports terms, a half marathon is a road race, also called non-stadia. Non-stadia events are sports events organized outside the stadium, including mountain, road, or sand races [2].

Two categories of participants compete in half marathons: amateur runners and performance runners. Amateurs are passionate runners who become competitive over time. Competitors are classified into age groups—18–34, 35–44, 45–54, 55–64, 65+ [3]—while master runners compete in the following categories, regardless of gender identity (men, women, non-binary): 35, 40, 45, 50, 55, 60, 65, 70, 75, 80, 85, 90, 95 years [4]. Participation rates and performance vary by age group.

Although not currently included in the Olympic Games, the half marathon has become popular among amateur runners in recent decades [5]. Its current popularity is driven by the large number of amateur runners, with an increasing number of women participating in such sports events [6]. A few studies shows that the number of women and men participating in half marathons and marathons increased significantly between 1999 and 2014, more so in half marathons and especially among women (123 times more women participate in half marathons than in marathons, and 75 times more men register for half marathons than for marathons) [5,6]. Similarly, in the USA, in 2019, the proportion of women (60%) surpassed that of men (40%) in half marathons, with more significant increases among master runners compared to younger runners [6]. Other studies also show that participation in competitions is relatively higher among women than men, especially in the 50+ age groups, with higher performance frequencies achieved at older ages [7,8,9,10].

In Romania, trends align with global ones, but the number of female participants in such races is still lower compared to other countries. The Romanian Athletics Federation (FRA) website indicates that the number of participants in the Bucharest Half Marathon has progressively increased since 2016, with a simultaneous increase in the number of female participants. Most women finished the race at the Cluj-Napoca Half Marathon from 2016–2022, with their numbers constantly growing, representing 29.06% of the total participants in 2022 [11].

Romanian master runners are registered with sports clubs affiliated with the FRA, also known as “veterans” [12] or senior runners [13]. As worldwide, in Romania, the scale and popularity of the half marathon are driven by amateurs, with fewer performance runners.

The increase in the number of endurance race participants and the diversification of participant groups (age, gender, profession, etc.) pose challenges in training design, monitoring, and execution, requiring adaptation to the specific needs of amateur runners. For women, training is conducted amidst numerous professional and family responsibilities [14]. Running many kilometers almost daily and weekly can interfere with their professional or family obligations or specific age-related needs. Additionally, women are more susceptible to injuries compared to men [15]. Training programs for master amateur runners should focus not only on physical performance but also on reducing physiological and relational stress. On the other hand, the heterogeneous profile of amateur half marathoners has led to diverse training models to adapt to the anatomical, physiological, or psychosocial factors influencing endurance runners’ performance and well-being, whether amateur or professional [10]. Unlike professional athletes, amateur half marathon runners—although they often have some prior running experience, most of them are not coached by a trainer—tend to exceed their upper physical capacity limits, frequently experiencing overtraining syndrome [16], lack of physiological adaptation [17], or deterioration of physical fitness [18,19]. The disadvantage of amateur runners is highlighted in other studies, showing that many performance-related factors and finish time predictions are under-researched [20,21]. Additionally, another drawback is the fact that systematic analyses of training plans primarily focus on professional runners [22], while those intended for amateur athletes are much rarer, though not entirely absent [10,20]. Under these conditions, although the half marathon as an event for amateurs has impressively developed, there is no consensus on the best training practices that would allow for the optimization of physical performance in a healthy manner [23]. However, there is a lot of empirical data that is not necessarily based on scientific studies [24]. As a result, controversies among specialists regarding the effectiveness of various training models and the need to adapt them to amateur runners’ particularities complicate the choice of optimal training type for a category or individual practitioners [25].

Sports training is a long-term activity carried out through a laborious process of planning and programming [26]. Training planning is a predictive process, based on experience and scientific knowledge, aimed at the rational, systematic, and sequential organization of training tasks and the recovery process to achieve performance objectives at specific times [27]. This involves paying special attention to the volume and intensity of effort, as these two variables are closely linked to the effectiveness and safety of training, directly influencing the capacity for adaptation and athletic progress [28]. The objective of training is to improve the athlete’s athletic skills and, ultimately, their performance level. The correct use of knowledge, experience, and principles of sports training leads to the design of effective training programs.

As revealed by a previous study that we conducted [29], many Romanian amateur athletes are interested in achieving faster race times and gaining social recognition for their efforts. In this context, they are concerned with optimizing their training efficiency. The importance of properly scheduling volume and intensity in this context is crucial, as it allows for the adjustment of training sessions to maximize performance without compromising the athletes’ health [27]. The same study showed that 55.9% of runners become injured during training or competitions or engage in demanding training exceeding 100 km per week, often followed by excessive fatigue, making it difficult to maintain motivation and diminishing running satisfaction. This further emphasizes the need to carefully regulate the volume and intensity of training to prevent overtraining and ensure proper recovery, which are essential for maintaining long-term physical and mental well-being [30]. Amateur female athletes have varied perceptions of effective training, lacking consensus on the optimal patterns of training methods, as well as on the volume and intensity of effort applied during each training phase at any given time [29]. In other words, it is important to consider that both the volume and intensity of effort are essential components of planning the sports training process, being fundamental to structuring an effective training program. Numerous training programs highlight various solutions for achieving performance. These training programs aim to develop economical training strategies for half marathoners, maintaining sports efficiency within optimal limits and positive effects on physical and mental health [31,32,33]. The impact of the strategies used is controversial, with evidence suggesting that moderate-intensity training enhances athletic performance similarly to high-intensity training. However, high-intensity training shows more significant differences in the physiological adaptation of the body to exertion [34,35].

Regarding the timing and scheduling of training programs, from the study of various sources, we have found that the time intervals for which these training plans are developed vary: 12/15/32 weeks [32,36], 12 weeks [37,38], 20 weeks [23], or 4–6 months [39]. Some believe that a one-year training program is essential [17]. However, verifying these training programs’ efficiency through experiments or quasi-experiments is rare or limited to elite runners [40].

As mentioned earlier, most training plan models target professional athletes, with far fewer tailored for amateur athletes. Generally, the traditional endurance training programming model is linear, featuring progressively structured training intervals in a predetermined sequence, including sessions, microcycles, and mesocycles that culminate in a macrocycle. Training periodization requires different training objectives, with its sequencing occurring during preparatory phases (focused on general and specific physical preparation, pre-competition, and competition preparation), concluding with a transition and recovery phase [28]. In the same context, another source emphasizes that scientifically approached training design and management should focus on key aspects of the training process, such as periodization, which require strategic adjustments to the volume and intensity of effort to optimize performance and physiological adaptation, along with training methods and monitoring, performance prediction, running technique, and the prevention and remediation of health issues associated with endurance running [23]. However, in practice, some half marathon runners employ a non-periodized strategy characterized by a fixed number of kilometers predetermined by repetitions throughout a training cycle [41]. This approach, while simplifying planning, may overlook critical aspects such as adjusting the volume and intensity of effort based on the athlete’s physical condition and specific goals, which can limit the optimization of performance and proper physiological adaptation.

In this complex context of planning and training practice for amateur female runners, our study aims to develop, apply, and confirm the efficiency of a staggered physical training program over one year (macrocycle) for female Master (+45) half marathoners, aiming to generalize the experience and good practices of nationally and internationally recognized runners adaptively. We believe their common training strategy can serve as a medium-term training model for amateur runners without a coach’s counseling benefits. The emphasis placed on volume and intensity in this plan highlights our belief that precise management of these dimensions is essential for achieving the desired sports performance, without compromising the health and well-being of the female athletes.

Based on these considerations, for the application of our annual physical training plan, we have formulated three research questions, each associated with a specific research direction. Question 1 (Q1): To what extent does the volume of effort performed by athletes align with the planned volume of effort in the annual physical training plan? The research direction for this question is to assess the congruence between planning and execution of training volume, evaluating the effectiveness of the programming. Question 2 (Q2): How does the intensity of effort performed by athletes compare with the proposed intensity of effort in the training plan for each mesocycle? This research direction aims to determine if the intensity of the training is appropriately adjusted to promote physiological adaptations without inducing risks of overtraining. Question 3 (Q3): Do the performances achieved in field tests meet the expectations established by the proposed average times in the training plan? The research direction for this question explores the relationship between scheduled preparation and actual performances, highlighting the importance of calibrating time goals according to the athletes’ real capabilities.

## 2. Materials and Methods

### 2.1. Study Design

The approach we implemented is ameliorative/formative in nature, as it aims to shape behavior [42], with the goal of improving or at least maintaining athletic performance in the half marathon event for master women (45+). Additionally, given that this is a physical training plan spread over the course of a year and considering that we have not found any macrocycle-type planning in the specialized literature, we consider our study to be a pilot one.

### 2.2. Study Subjects and Research Team

Within the study, female master athletes aged 45 and over were selected because, as mentioned earlier, several studies indicate that their participation in competitions has increased significantly globally over the past three decades [43,44,45]. From an age perspective, different age categories are considered to ensure some heterogeneity of the sample and, consequently, greater internal validity of research [42].

The subject group includes six female amateur runners (S1–S6) who practice long-distance running both indoor and outdoor, road and mountain running, and fall into the 45–49 and 50+ age categories. They are Master runners registered with Romanian sports clubs affiliated with the FRA, and at the study’s outset, they had over 10 years of endurance running experience (Table 1). Being amateur runners, they do not have personal coaches. All participants live in urban areas and have different professions. The profession of the participants includes three physical education teachers, one doctor, one marketing specialist, and one museum curator. These characteristics were inclusion criteria, while health status and injuries were exclusion criteria.

For this study, the Romanian female runners (S1–S6) were recruited at the World Masters Mountain Running Championships (Telfes, Austria 2021), where the Romanian team, including these female runners, placed third after Italy and Germany. In 2021, all six female runners were active, participating in national and international competitions, with one (S5) also participating in two virtual competitions.

To assess the health status of the six female runners, they underwent an initial laboratory diagnosis (in the first part of mesocycle 1; 4–31 October 2021) at the SZJA Sportlab Center in Cârța, Harghita County [46]. The specialized anamnesis showed no cardio-respiratory pathologies or injury history in the last six months. To determine the female runners’ maximum heart rate (MHR) for establishing the training intensity and training zones, an effort test was conducted using a professional treadmill (HP Cosmos—pulsar^®^3p, h/p/cosmos sports & medical gmbh/Nußdorf, Germany) following the Brucea protocol at the same location [47]. The HP Cosmos treadmill is equipped with a chest strap connected to an external monitoring system, which displays the data on the screen in real time.

As study coordinators (the research team), five faculty members from the Faculty of Physical Education and Mountain Sports, Transilvania University of Brașov, participated in designing PASm-12, approving and adjusting its content as needed and monitoring the training process of the six female runners. Our research approach was approved by the Department of Physical Education and Special Motricity of the faculty (no. 332/1/23.09.2021, approved on 23 September 2021) and was based on the Helsinki Declaration regarding research ethics, ensuring the rights, safety, and well-being of the participants. Informed consent of the participants was obtained. Additionally, during the implementation of PASm-12, periodic consultation was sought from a sports nutrition specialist (Superfit Center in Bucharest) [48].

### 2.3. Procedure

Throughout the training program, periodic non-specific and specific tests were applied to the six female runners for more rigorous control and regulation of the staggered physical training process over one year.

The independent variable consists of the content of the annual physical training program for Masters +45 half marathoners (PASm-12) and was largely followed independently (self-training) by each study participant through a personalized training program derived from PASm-12, monitored, and staggered over 12 training mesocycles. Since PASm-12 is applied in the runners’ natural environment, the control of the independent variable is not very strict, but the research has the advantage of being an ecological intervention, making its results and conclusions more suitable for practical problem-solving compared to laboratory experiments [49].

Dependent variables include the participants’ performances in applying the dependent variable, i.e., running distances achieved, the intensities at which they were performed, with both effort indicators reported to the corresponding values proposed by PASm-12. Additionally, partial or complete test times in five field tests conducted in non-competitive and competitive environments, where the runners gathered at predetermined locations during PASm-12 design, also represented elements of the dependent variable.

After selecting the subject group, participants were informed about the study’s general objectives and the annual physical training program’s structure and content—as well as the competition calendar (2021–2022)—were agreed upon, with potential adjustments depending on the evaluations/results obtained from the scheduled/designed tests.

#### 2.3.1. The Presentation of the Annual Physical Training Plan for the Half Marathon (PASm-12)

Considering various training models and debates regarding the advantages or disadvantages of certain periodization methods, as well as the consequences of different training volumes (kilometers covered) and intensities (execution times/tempo, expressed as a percentage of maximum effort capacity per subject) on performance and maintaining female runners’ health, we opted for a traditional linear hierarchical training model in our study. We chose this training–planning strategy given the selected female runners’ already-developed motor skills (technical), appropriate physical preparation level, and well-defined training routines.

As structured, PASm-12 spans 12 mesocycle comprising 52 microcycles, each microcycle consisting of 4 or 5 training sessions per week, depending on the training period. The methodology for planning the annual physical training plan for women Master 45+ runners, covering the period 4 October 2021–30 September 2022, proposes a progressive, cross-sectional arrangement of cycles/periods/phases of training [27,28,30]. Structurally, PASm-12 features a preparatory period (PP 4 October 2021–30 January 2022) developed into two phases: general physical preparation (GPP, 4–31 October 2021) and specific physical preparation (SPP, 1 November 2021–30 January 2022). This is followed by a pre-competition period (PCP, 31 January–3 April 2022), a competition period (CP, 4 April–18 September 2022), and concludes with a transition period (TP, 19–30 September 2022). In our planning model, training volume and intensity increase gradually in GPP, while in SPP and PCP, volume decreases to medium, and intensity increases to an optimal level according to event-specific demands. In CP, intensity fluctuates from high (submaximal) to optimal for the half marathon event, while volume fluctuates from high to medium, depending on runners’ performance objectives related to the significance of tests/competitions in the pre-set calendar. PASm-12 is structured to allow the runners’ functional recovery in a permanent and controlled manner as they progress toward the main period (CP) and the major objective competition [30,50].

The plan initially proposes moderate training intensity (about 70% of the maximum heart rate per athlete) and relatively small training volume (about 40 km/week), with indicators progressively increasing during the training process. Training sessions were planned to allow physiological recovery for participants and facilitate the body’s functional adaptation to effort.

For the agreed-upon preparation period, a unanimously agreed competition calendar was developed, correlated with PASm-12’s quantitative and qualitative indicators (volume/intensity). The collaborative development of the competition calendar is justified by the need to harmonize runners’ professional, family, and financial activities. Thus, the competition calendar included five field tests (Table 2), with the final one representing an official competition, termed the major objective.

For each of these tests, the PASm-12 pre-established the race time (referred to by us as the “proposed running time”) with a tempo/km range set between a minimum and maximum limit, aiming to achieve an average reference value. These times were fixed as an anticipated consequence of the training plan’s content periods. Thus, for the Zărnești test, scheduled in mesocycle IV/microcycle 17 (3–30 January 2022), a running tempo for the 10 km distance was set at 5′10″/km (maximum limit, slower time)–4′50″/km (minimum limit, faster time), equating to 3100 s/10 km and 2900 s/10 km respectively, with an average between the two values of 3000 s/10 km (50 min). For the other 4 tests—scheduled in Cluj-Napoca—mesocycle V/microcycle 19 (7–13 February 2022), Oradea—mesocycle VI/microcycle 24 (14–20 March 2022), Brașov—mesocycle VII/microcycle 28 (11–17 April 2022)—a running tempo for the regulation distance of the half marathon was projected between 5′40″/km (maximum) and 5′30″/km (minimum), resulting in 7140 s maximum/21 km and 6930 s minimum/21 km, with an average test time of 7035 s/21 km (117 min and 15 s). For EMACNS—Grosseto—mesocycle VIII/microcycle 32 (9–15 May 2022), a running tempo of 5′10″/km (maximum) and 4′50″/km (minimum) was established, with a maximum time for completing the full race distance of 6510 s/21 km and a minimum of 6090 s/21 km. The proposed finishing average time (finish time) for the race was 6300 s/21 km (105 min).

Macroindicators for PASm-12 (training days, mesocycles, microcycles, number of field tests, laboratory tests) are presented in Table 3. In terms of content and training means, it should be noted that some microcycles in the plan are identical, while others vary based on the training objectives assigned to each mesocycle.

**Figure 1:**
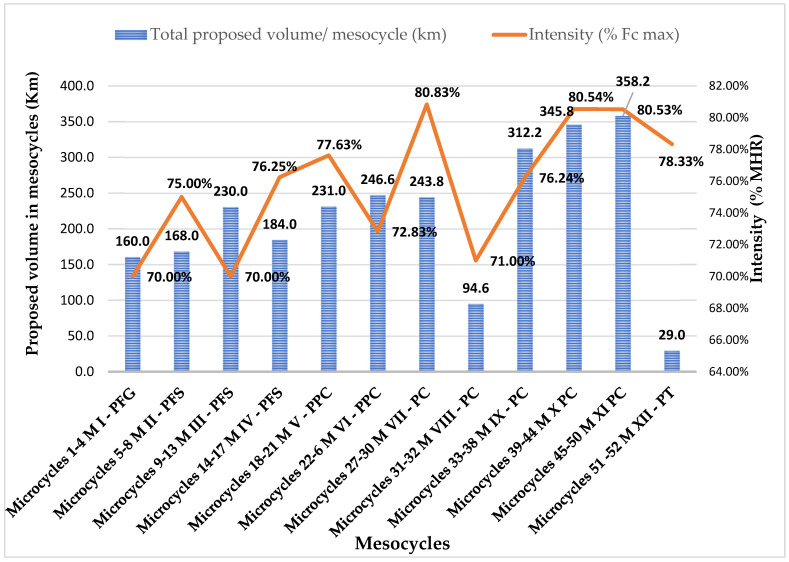
Dynamics of proposed training volume and intensity—average values/mesocycle in PASm-12.

For an overview of the programmed means in PASm-12 and the volume indicators (distance covered/km, execution duration), as well as the intensity of effort (average percentage intensity of effort/mesocycles) predetermined for the plan, we summarize some relevant characteristics in Table 4 and the dynamics of the proposed volume and intensity distribution in Figure 1.

#### 2.3.2. Remote Monitoring Equipment for Runners’ Activity

A necessary step in conducting our study, which was largely monitored remotely, involved the acquisition of six Garmin Fenix 6S PRO watches (Garmin Ltd., Olathe, KS, USA) with GPS technology. Currently, a growing number of running enthusiasts use technology for self-monitoring. An online study with a sample of 3723 participants analyzed how endurance runners use technology for training monitoring [51]. The results indicate that approximately 6 out of 10 respondents (59.5%) used a sports watch, 28.4% used a running app, and only 12.1% did not use either a watch or a running app. The watches were used to monitor distance (96.20%), time (90.0%), speed (85.5%), and calories (8.8%). Another study indicates that, although Garmin Fenix 6 watches may have a tendency to underestimate HR at low intensities and overestimate it at high intensities, they still provide useful and relevant data, especially in contexts where continuous monitoring and portability are essential [52]. In our study, throughout the entire application period of PASm-12, all training indicators considered relevant—training days and hours, distance covered, volume parameters per athlete (distances, effort durations), and effort intensity per athlete (pace, heart rate, etc.)—were continuously monitored and centralized weekly via Bluetooth by the research team for each athlete through the data provided by the Garmin Connect (Version 5.5) app. The watches were acquired through the “Life Quality and Human Performance” Research Laboratory of the Faculty of Physical Education and Mountain Sports in Brașov.

### 2.4. Statistical Analyses

The collected data were analyzed using SPSS v.23 for Windows. The significance level was set at *p* ≤ 0.05. Descriptive data are reported as mean and standard deviation (SD) and sometimes as percentages (%). Differences in continuous variables (volume, intensity, tempo, test execution times) were analyzed using the paired *t*-test. Given the small sample size, we used bootstrapping, where the statistical software creates simulated samples similar to the analyzed one, increasing result accuracy and stability. The ideal is to use 1000 samples, which we applied in our data processing [42].

In addition, we calculated the effect size using two distinct methods, tailored to the specific context of each indicator, with the aim of ensuring a rigorous and appropriate evaluation of the obtained data. Thus, for the indicators of effort volume and intensity, we chose to apply Cohen’s f^2^, as this method allows for a comprehensive assessment of the variance explained by our training model in relation to the proposed volume and intensity. For the comparison between the proposed and achieved times in the five tests, we opted for the Cohen’s d formula, which is suitable for measuring the differences between two means, taking into account the standard deviation, thereby ensuring an accurate evaluation of individual performances against the expected values. The effect size serves to evaluate the impact of the intervention and to facilitate the justification of the necessary number of participants in future studies as well as to estimate the magnitude of the differences that other researchers might expect [42].

## 3. Results

### 3.1. The Level of Fulfillment of the Annual Physical Training Plan for Half Marathon Runners Aged 45+—PASm-12

Compared to what was proposed in PASm-12, the six research subjects reported the volume and intensity indicators they actually achieved in varying proportions. The following results will be presented accordingly: effort volume, determination of maximum heart rate (MHR) and establishment of training zones, as well as effort intensity and the testing times obtained.

#### 3.1.1. Comparison of Proposed and Realized Training Volume through PASm-12 (Q1)

In Table 5, the data show that athlete S3 achieved the highest training volume (2478 km) while athlete S1 achieved the lowest (2232 km). These data allow us to assert that PASm-12 was not fully applied, only at 90.2%.

**Table 5:** Effort volume realized by runners and proposed by PASm-12.

Mesocycles	Effort Volume Realized per Athlete (S)/km						Proposed Training Volume/km	
	S1	S2	S3	S4	S5	S6		
I	132	156	160	130	148	142	160.00	
II	146	150	162	142	160	136	168.00	
III	162	162	178	158	170	172	230.00	
IV	172	178	180	168	176	168	184.00	
V	178	202	220	185	206	201	231.00	
VI	212	230	236	228	232	240	246.60	
VII	228	227	230	228	230	235	243.80	
VIII	90	150	95	90	93	95	94.60	
IX	302	320	332	308	280	310	312.20	
X	310	320	340	300	298	345	345.80	
XI	270	308	315	290	265	310	358.20	
XII	30 *	50 *	30 *	20 *	40 *	20 *	29.00 *	
Total effort volume achieved per athlete (km)	2232	2453	2478	2247	2298	2374		2603.2
							2347	
Effort volume achieved per athlete from the proposed effort volume (%)	8.7	94.2	95.2	86.3	88.3	91.2	90.2	

* Note: In the proposed volume value, the athletes also accounted for mountain hiking activities to a greater or lesser extent. To verify if the proposed training volume in each mesocycle is statistically equivalent to the training volume performed (Q1), we used the paired *t*-test to compare the means of the two training volumes (proposed/realized) across the sample/12 mesocycle, employing the bootstrapping method for small samples (Table 6).

**Table 6:** Differences between realized and proposed training volume through PASm-12.

Pair	Volume Indicator Variable Compared/Mesocycle/Realized-Proposed	Mean S1–S6 (km)	Difference	t	*p*
1	Realized training volume mesocycle I	144.7	−15.33	−3.052	0.028
	Proposed training volume mesocycle I	160.00			
2	Realized training volume mesocycle II	149.33	−18.67	−4.495	0.006
	Proposed training volume mesocycle II	168.0			
3	Realized training volume mesocycle III	167.00	−63.00	−20.40	0.001
	Proposed training volume mesocycle III	230.00			
4	Realized training volume mesocycle IV	173.67	−10.33	−4.939	0.004
	Proposed training volume mesocycle IV	184.00			
5	Realized training volume mesocycle V	198.67	−32.33	−5.247	0.003
	Proposed training volume mesocycle V	231.00			
6	Realized training volume mesocycle VI	229.67	−16.93	−4.290	0.008
	Proposed training volume mesocycle VI	246.60			
7	Realized training volume mesocycle VII	229.67	−14.13	−12.041	0.001
	Proposed training volume mesocycle VII	243.08			
8	Realized training volume mesocycle VIII	102.17	7.57	0.787	0.467
	Proposed training volume mesocycle VIII	94.60			
9	Realized training volume mesocycle IX	308.67	−3.53	−0.493	0.643
	Proposed training volume mesocycle IX	312.20			
10	Realized training volume mesocycle X	318.83	−26.97	−3.302	0.021
	Proposed training volume mesocycle X	345.80			
11	Realized training volume mesocycle XI	293.00	−65.20	−7.414	0.001
	Proposed training volume mesocycle XI	358.20			
12	Realized training volume mesocycle XII	31.67	2.67	0.559	0.600
	Proposed training volume mesocycle XII	29.00			
Total effort volume achieved (macrocycles)		2347.00	−256.2	−5.997	0.002
Total proposed effort volume (macrocycles)		2603.20			

If the significance of the comparison test is greater than *p* = 0.05, we accept that the data do not provide sufficient evidence to conclude that the volume of effort performed by the athletes aligns with the planned volume of effort in the annual physical training plan. On the other hand, if *p* ≤ 0.05, we reject the null hypothesis, suggesting that there is sufficient evidence to conclude that the volume of effort performed by the athletes is congruent with the planned volume, indicating the effectiveness of the scheduling.

Regarding the effect size, the calculated R^2^ value is 0.8619, indicating that 86.19% of the variation in the realized training volume can be explained by the proposed training volume through PASm-12. This shows a strong correlation between the two sets of data. Additionally, the Cohen’s f^2^ value for the mean difference in training volume is approximately 6.24, indicating a large effect size. This suggests that the difference between the proposed and realized training volume is not only statistically significant but also has a substantial practical impact.

#### 3.1.2. Determination of Maximum Heart Rate (MHR) and Establishment of Training Zones for the Research Subjects

Based on the laboratory tests presented in the procedure section, individual training zones were established for each athlete, including maximum heart rate (MHR), which are essential for monitoring and adjusting the intensity of effort during the application of PASm-12 (Table 7).

Based on initial laboratory data, we proposed and then monitored PASm-12’s training intensity for each athlete. Each mesocycle and microcycle had pre-set training intensity values (proposed values) applied in practice (realized values) through PASm-12.

#### 3.1.3. Comparison of Proposed and Realized Training Intensity through PASm-12 (Q2)

The intensity indicator considered was based on the physiological response of the body to the strength of the stimulus/the physical exercise performed, specifically reflected by the heart rate (HR), expressed as the average percentage per mesocycle per athlete. This percentage value is related to the maximum heart rate (MHR) of each athlete (Table 8).

These values, taken as the average percentage values of realized training intensity by S1–S6/mesocycle (relative to MHR/S), were compared with the proposed percentage values of mesocycle averages by PASm-12 (Table 9, Figure 1). Data analysis shows that training intensity was realized at 94.8%, indicating almost complete plan application.

To verify if the proposed training intensity/mesocycle is statistically equivalent to the realized training intensity (Q2), we used the same statistical comparison procedure of the two effort intensities’ means (realized/proposed) for the entire sample S1–S6/12 mesocycle, using the paired *t*-test and the bootstrapping method (1000 samples) (Table 9).

We observe that, similar to the training volume, the realized training intensity by runners (average value S1–S6/mesocycle) is generally lower than the proposed intensity, with mostly negative differences. However, there are some exceptions where the realized intensity is close to or even higher than the proposed (mesocycle V, IX, and XII). The significance of differences is statistically significant for most mesocycle, except for mesocycle I, II, V, VIII, IX, and XII, where differences are not statistically significant. We conclude that the statistical differences were insignificant in most mesocycle, indicating good conformity with the initial plan (PASm-12).

By calculating the effect size, the R^2^ value is 0.314, indicating that 31.4% of the variation in the dependent variable (realized effort intensity) can be explained by the independent variable, namely the proposed effort intensity through PASm-12. This is a relatively moderate coefficient, indicating a significant, but not very strong, relationship between the variables. However, the Cohen’s f^2^ value is 0.45, which indicates a large effect, and the independent variable (PASm-12) has a substantial impact on the dependent variable (average intensities per mesocycle/S1–S6). This suggests that the plan was robust enough to guide the athletes towards achieving most of the proposed objectives, also demonstrating that, even with deviations from the initial plan, PASm-12 managed to maintain a coherent direction in our female athletes’ training.

#### 3.1.4. Comparison of Realized Test Times with Proposed Execution Times in PASm-12 (Q3)

To answer the question: How are runners’ performances influenced by the partial application (90.2% of the proposed training volume and 94.8% of the requested intensity) of PASm-12?. We chose to compare the realized test times of runners in the five tests with the proposed times in the microcycle within the mesocycle corresponding to the testing moments in PASm-12. We started from the premise that if these compared time values do not indicate significant statistical differences, and the test execution times and those proposed by PASm-12 are statistically equivalent, we can affirm that the training volume and intensity realized by runners are sufficient to achieve the planned performance goals for the major objective competition (EMACNS Grosseto).

**Figure 2:**
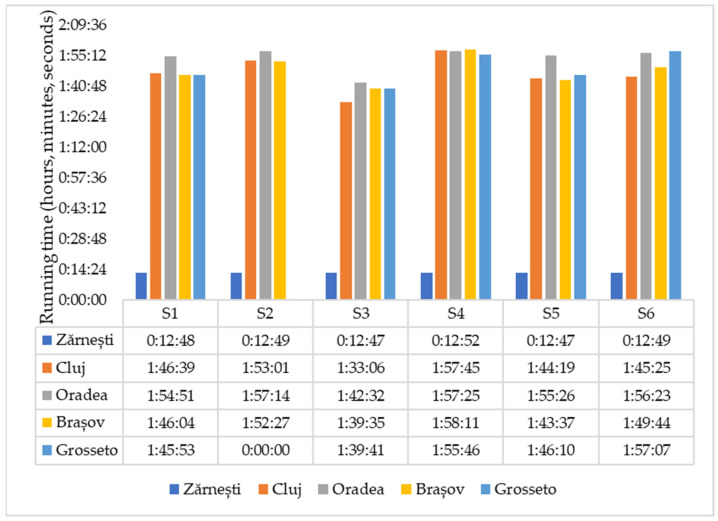
Results of the five tests in the 2020–2021 competition year.

Results expressed in race times in the five tests conducted by the six female runners are presented in Figure 2. The female runners participated in all tests (except S2 in test 5), namely the non-specific 10 km test and four specific 21 km tests. In PASm-12, for the non-specific test (Zărnești 10 km), the proposed and initially accepted tempo by runners was 5′10–4′50″/km, with an average of 5 min/km, converting to 3000 s/10 km for the entire test. For the specific tests in Cluj-Napoca, Oradea, and Brașov (21 Km), the proposed tempo by PASm-12 for the entire half marathon was 5′40″–5′30″/km, with an average tempo of 5′35″/km, equivalent to 7035 s/21 km. For the European Masters Athletics Championships Non-Stadia (EMACNS Grosseto—Italy), the proposed training program tempo was 5′10″–4′50″/km, with an average of 5′/km—thus, 6300 s/21 km.

To verify Q3, we used the paired *t*-test to compare the realized test times (average value/test S1–S6) with the proposed average times per km (running tempo/km) at the five testing moments included in PASm-12. Calculations show that these times, except for the Grosseto test, are shorter, thus better, compared to the projected average test times in PASm-12 in the corresponding mesocycle/microcycle. Four differences between the two series of times are positive, and the time obtained at EMACNS—Grosseto (Italy) is longer than the proposed time, with a negative but statistically insignificant difference (Table 10).

In the case of proposed average times (s/km/test) as a performance benchmark in executing the tests, analysis of these differences indicates varied results in testing: for three tests (Zărnești, Oradea, and Grosseto), the average differences are not significant (*p* > 0.05), meaning the runners achieved the overall “finish time” indicator at the projected average value set by PASm-12. For the tests in Cluj-Napoca and Brașov, the average differences are statistically significant, with *p* being less than 0.05; in these tests, the runners had weaker overall performance (S1–S6) than proposed by PASm-12. Therefore, it cannot be concluded with certainty regarding achieving the “average test times/km” proposed in PASm-12 and the actual test times, taken as an average value for the six runners in executing the tests.

The effect size for each test, specifically the differences between the planned times and the actual times achieved, is as follows: for the 10 km test in Zărnești, Cohen’s d was 0.44, indicating a small to moderate difference; for the 21 km test in Cluj-Napoca, Cohen’s d was 1.68, indicating a large difference, favorable to the actual times achieved; for the 21 km test in Oradea, Cohen’s d was 0.41, indicating a small difference, with actual times close to those planned; for the 21 km test in Brașov, Cohen’s d was 1.39, showing a large difference, favorable to the actual times achieved, which were shorter; and for the 21 km test in Grosseto, Cohen’s d was −0.53, suggesting a moderate difference, with the actual times being longer than those planned.

After executing the main test—the objective (EMACNS—Grosseto), the entire group agreed to continue monitoring the training process through PASm-12 until its full completion without participating in common competitions imposed by PASm-12. This request was largely due to family and professional responsibilities and financial constraints. However, all runners agreed to participate in the study until the full execution of PASm-12 contents and to report continuously via smartwatches both the requested training parameters and competition participation within the competition period defined by PASm-12, even if these were not always exclusively related to the half marathon event. Data collected from S1–S6 show that female runners continued competitive activity (except S1—due to professional and academic reasons), participating in both national and international competitions (Table 11).

From these quantitative data, it is observed that the participation of each subject varies in number (between 0–10), with the predominant ones being at nationally scheduled competitions.

## 4. Discussion

The purpose of this study was to design and implement an annual physical training plan for master athletes aged 45 and above, specifically focusing on the half marathon (PASm-12) event. This age category generally includes amateur practitioners who often lack a scientifically grounded training program, at least as a guiding framework, to meet the multiple demands associated with this event. Moreover, they often do not benefit from the counseling of a specialized coach to coordinate the complex aspects of sports training and continuously adapt training methods and means to the efficiency and safety requirements necessary for the event. To apply and validate such a medium-term (over one calendar year) plan, we involved six Master amateur runners (+45 years), each with at least 10 years of competitive experience. We considered their experience and competition results prior to the study as a guarantee for both good practice of the specialized training process within our approach and future confidence from other amateur runners regarding the sustainability of the sports training model followed by our athletes.

### 4.1. Discussions on the Structure, Content, and Effort Indicators of PASm-12 Compared to Other Planning Documents Presented in the Specialized Literature

Compared to what is mentioned in Section 2.3.1, other studies applying endurance event (half marathon, marathon) training programming based on the same linear planning model present somewhat simplified periodization strategies compared to PASm-12. For example, planning sequences include four stages with a preparatory period (November to February), a pre-competition period (March to the end of April), and a competition period (May to September) [22] or a single 20-week competition macrocycle (“evolutionary periodization”) following a 3-month preparatory period. In this case, the competition macrocycle is divided into two mesocycles, each of 10 weeks, with the training process applied to a single performance athlete aged 50 [53]. Another planning method on a relatively short term involves a three-phase plan—base phase, training phase, and recovery phase [54]—or recommends 4–6 months of preparation until competition day [39]. Another study suggests a 12-week preparation plan for an experienced athlete [37]. In planning PASm-12, we considered a one-year training program to provide a comprehensive strategy for training and allow the practitioner to form a continuous anticipatory behavior focused on the provided methodology.

From the perspective of the number of training sessions, in PASm-12 we proposed that the amateur athletes undertake four or five training sessions per week. In other training models—for example, for the marathon distance—11–13 training sessions have been scheduled for high-performance athletes [22]. For the half marathon distance, other sources suggest either 5–6 training sessions per week for a 50-year-old performance athlete [53], 6 training sessions per week for advanced half marathoners [39], or 5 weekly training sessions with 2 rest days [54].

The means/exercises most commonly used by half marathon runners, according to studies focused on this direction, include easy running, alternating walking and easy running, acceleration runs, repeated sprint training, aerobic–anaerobic threshold running, progressive reduction in distance running, aerobic threshold long runs, Fartlek, steady-tempo running, interval running, strength endurance training—circuit type, jumps, etc. [32,35,36,39,53,54,55,56]. In PASm-12, similar means/exercises were largely utilized. In summary, we applied steady-tempo running at a pace of 5′40″–5′30″/km (the most frequently used specific method in PASm-12, representing 35.1% of total means used), moderate-paced long-distance running (making up 26.9% of the total), interval running (9.5%), Fartlek running (9% of total training means), steady-tempo running at 5′10″–4′50″/km (programmed at 5.8%), whereas hill running and steady-tempo running at 5′40″–5′10″/km were the least used exercises in PASm-12, representing 5.6% and 4.6% of the total means programmed, respectively. Additionally, competitive races conducted as tests in PASm-12 account for 3.6%. In contrast to PASm-12, another study recommends replacing 25–30% of running volume with other training options [54], which is agreed upon in our case.

#### The Dynamics of Volume and Intensity Indicators in PASm-12

The most important training indicators targeted by PASm-12 were the volume and intensity of effort. Quantitative parameters—volume indicators such as running distance and duration (when applicable)—were considered, while execution times, related with the predetermined tempo and the athlete’s functional demand (heart rate relative to maximum heart rate—MHR), provided data on the qualitative component of effort, i.e., its intensity (the functional demand of the body in response to effort). In PASm-12, during the preparatory period (PP, October–January/mesocycle I–IV), we proposed a volume of 160 km in GPP and 194.00 ± 32.18 km in SPP, with runners running an average of 40–46 km per week, 4 times per week, at an average intensity of up to 70% (72.8%) of MHR/athlete (monitored via Garmin watches). In the pre-competitive period (PPC, February–March/mesocycle V–VI), a larger running volume of 238.8 ± 11.03 km was proposed, to be completed over 5 training sessions per week, with weekly volumes ranging from 36 km to 64.8 km and intensities between 65% and 82% of each athlete’s capabilities (with an average of 75.2%/PPC mesocycle). During the competitive period (CP, April to mid-September/mesocycle VII–XI), the proposed training volume was 270.92 ± 108.11 km, with runners running 42–67 km per week at intensity levels ranging from 68% to 85% of MHR/athlete (average of 77.8%). In the transition period (TP, mid-September, mesocycle XII), a volume of 29 km was proposed, with weekly training intensity ranging from 70% to 77% of MHR/athlete, averaging 78.2% per mesocycle. Our effort parameter phasing strategy in PASm-12 is similarly noted in other studies. For example, for an experienced half marathoner, weekly running volumes of 32, 48, or 64 km are recommended [37], and for training intensity, combining moderate- and high-intensity sessions is considered an effective approach [40]. In another study on endurance runners, combining volume and intensity of effort showed no consensus on the most effective practices for maintaining or developing health and performance in amateur runners: either increase intensity by 10% per week or run 70 km per week [53]. In PASm-12, we aimed to facilitate progressive biological adaptation to effort by scheduling shorter distances and times at the beginning of the training period to prevent early dropouts, often justified by amateur runners due to lack of time, injuries, or dissatisfaction with training due to fatigue [29]. Running more than 65 km per week for men and between 48–63 km per week for women is associated with higher health risks for amateur runners [57,58,59], and preventing these risks involves reducing training volume [60]. Excessive training volume can be detrimental, leading to fatigue, reduced training efficiency, uneconomic muscle effort, and increased injury risk [17]. Some authors, however, find current research results inconclusive in this regard [61].

### 4.2. Discussions on Comparison of Effort Volume and Intensity Indicators Proposed by PASm-12 and Those Achieved by S1–S6

#### 4.2.1. Comparison of Proposed and Achieved Volume—The Response to Q

To analyze the implementation of PASm-12 in terms of volume, we compared the total running volume completed by the six runners (2347 km) with the volume proposed by the annual plan (2603.2 km). The comparison shows a difference of 256.2 km between the proposed and achieved volumes over the macrocycle, corresponding to a 90.2% implementation rate of PASm-12 (Table 5). Generally, the female runners ran less than the proposed volume, with differences mostly negative and only twice positive in mesocycle VIII and XII. The differences are statistically significant at *p* = 0.05 for most mesocycle, except for VIII, IX, and XII (Table 6). Thus, we accept that for mesocycles VIII, IX, and XII, the differences between the proposed and the actual volume of effort are not statistically significant. In other words, in these three mesocycles, the athletes ran, on average, distances statistically equivalent to those proposed in PASm-12. Conversely, we reject that the differences between the proposed and actual volume are not statistically significant for the other mesocycles, where the proposed volume is greater than what was actually achieved. Therefore, we can state that the annual training plan (PASm-12), from the perspective of running volume, was implemented, but not fully. Periodic reports show that the overall training volume was achieved by the runners as follows: 85.7% by S1; 94.2% by S2; 95.2% by S3; 86.3% by S4; 88.3% by S5; and 91.2% by S6.

The analysis of the effect size for the observed differences in the training volume achieved compared to the proposed one highlighted a significant effect size (Cohen’s f^2^ = 6.24). The large effect size indicates that variations in training volume are sufficiently pronounced to have observable consequences on performance and athletes’ adaptation. This finding emphasizes not only the statistical significance and substantial practical impact of the differences but also their relevance in the context of training planning for female master 45+ amateur athletes. Furthermore, the importance of this effect size extends beyond the validation of the PASm-12 training program, serving as a guiding tool for researchers planning similar studies and contributing to the improvement of reliability and validity in future research on this area.

#### 4.2.2. Comparison of the Intensity Percentage Value Proposed in PASm-12 and That Achieved by S1–S6—The Response to Q2

The training intensity achieved by the runners was implemented on average (S1–S6) at 94.79% of the proposed PASm-12 intensity. This high percentage indicates that although the achieved intensity (mesocycle I–XII/S1–S6: 69%; 70%; 68%; 69%; 73%; 69%; 76%; 69%; 74%; 77%; 76%; 71%) was slightly lower than the proposed intensity (70.0%; 75.0%; 70.0%; 76.3%; 77.6%; 72.8%; 80.8%; 71.0%; 76.2%; 80.5%; 80.5%; 78.3%) in most mesocycles, the annual training program implementation was very close to the established objectives. Thus, we accept that for mesocycles I, II, V, VIII, IX, and XII, the actual intensity of effort is statistically equivalent to the proposed intensity of effort. Conversely, we reject that the actual intensity of effort is equivalent to the proposed intensity for the other mesocycles, where the proposed intensity is significantly higher than what was actually achieved. Therefore, while PASm-12 was largely followed in terms of training intensity, it was not fully implemented (94.8%), indicating the need for some adjustments in future applications to ensure that the proposed intensity is met in all mesocycle.

The results of the effect size calculation and the coefficient of determination (R^2^) highlight several important aspects in evaluating the effectiveness of the PASm-12 plan, especially in the context of self-managed training and athletes’ self-reporting of data. With an R^2^ of 0.314 and Cohen’s f^2^ of 0.45, it can be said that the independent variable (the training intensity proposed by PASm-12) explains a moderate portion of the variation in the achieved training intensity and has a large effect on this variable. This means that the proposed training intensity plays a significant role in determining the achieved training intensity. Although the R^2^ coefficient, calculated at 0.314, indicates a discrepancy between the proposed and achieved values, it should be emphasized that this does not necessarily represent a limitation of the PASm-12 plan itself but rather reflects the inherent variability in a long-term training program involving athletes who self-manage their training. This variability actually demonstrates the flexibility of the PASm-12 plan, which allowed for personalized adaptation of the training to the needs and capabilities of each athlete without compromising the overall objectives of the plan.

In principle, for both indicators of effort analyzed, we agree with studies that claim that a high training volume and a high training pace (intensity) are predictors of a better race finish time [57,59,62,63].

#### 4.2.3. Comparison of Test Results and Proposed Times in PASm-12 (The Response to Q3)

We aimed to assess the efficiency of PASm-12, considering that it was not fully implemented (as expected for an intervention conducted under natural conditions over a relatively long period). We compared the average times per km from the field tests of the six athletes to the average time per test proposed by PASm-12. If these comparisons show no significant statistical differences, indicating the times achieved and proposed are statistically equivalent, we can affirm that the achieved volume and intensity of effort are sufficient to meet the planned performance. Following data analysis, we consider the results satisfactory, as they indicate that most of the times achieved in the field are lower than the average times proposed by PASm-12 (Table 9). Corroborating the results obtained from the responses to Q3, it can be asserted that PASm-12 is suitable for training for the half marathon, as the athletes’ performances in the five tests were either better than or equivalent to the proposed times. Compared to the average proposed time per test, which is between the maximum and minimum (Table 9), the times obtained per test (average value S1–S6) are as follows: four of the recorded times are lower than the proposed time (with two differences being statistically significantly smaller), and the time recorded in Grosseto is weaker, but the difference is statistically insignificant. Thus, we consider the times to be equivalent.

From the perspective of effect sizes for the times achieved compared to those proposed across the five tests, a valuable insight into the actual effectiveness of the PASm-12 plan can be drawn. The variation in effect sizes among the five tests reflects the direct impact of the plan on performance, highlighting the athletes’ ability to adapt and respond to the specific demands of competitions. For most tests, except Grosseto, effect sizes (Cohen’s d) indicate improved performance, where athletes achieved better times than those proposed. This demonstrates the effectiveness of PASm-12 in preparing athletes for competitions, validating its scientific and tailored approach to master athletes. A notable exception was the Grosseto test, where the negative effect size (−0.53) suggests that performance was below expectations. This finding indicates that competition performance is often influenced by a range of external factors related to the competitive environment. Factors such as course conditions, psychological pressure, and competition dynamics can affect results, causing them to differ from those obtained in non-competitive tests. This variation necessitates a deeper analysis of the factors that might be responsible for this deviation.

As such, although the indicators of training volume and intensity were adhered to only 90.2% and 94.8% throughout the entire macrocycle/year of preparation as outlined in PASm-12, the female athletes managed to perform unequivocally at the average level accepted by consensus and projected by PASm-12. Therefore, following PASm-12 to a large extent results in positive outcomes for the performance of the female athletes in our subject group, even under conditions of international competition. This fact is also demonstrated by the placements achieved at the target competition set by PASm-12 (European Masters Athletics Championships Non Stadia, held in Grosseto, Italy, from 12–15 May 2022), where our female athletes achieved the following rankings: S1 finished 6th (W50), recording her personal best time of 01:45:52 (PB) during the study period; S2 did not participate in the EMACNS—Grosseto due to family issues; S3 recorded a time of 01:39:41, placing 3rd (W45); S4 completed the race with a time of 01:55:46, placing 8th (W55); S5 achieved a time of 01:46:10, finishing 12th (W50); and S6 obtained a time of 01:57:07, placing 17th (W45).

In conclusion, we believe that the study results underscore the potential of the PASm-12 plan to serve as an effective model for preparing master female athletes for half marathon events, encouraging continuous adaptation and adjustment of training plans based on real feedback obtained through testing. This approach not only enhances performance but also improves athletes’ motivation and satisfaction, which are essential for sustaining long-term commitment to the sport.

### 4.3. Limitations

Despite these positive results supporting the practical viability of PASm-12, our study has limitations. The primary limitation is the small sample size, resulting from the difficulty of finding participants willing to follow a common training program over a year through self-instruction. This small sample size may affect the generalizability of the results. It is possible that the use of a control group may have contributed to the reporting of more relevant data. We attempted to mitigate this by using the statistical bootstrapping method, as is done in case studies [42,49]. Another limitation is the use of self-reported data by the athletes, which can negatively affect objectivity and, consequently, the quality of the research. However, this method of data transfer was the only feasible way to explore the training indicators of interest. Additionally, there are opinions suggesting that the accuracy of measurements performed by smartwatches, a procedure we also adopted, is influenced by errors, varying in magnitude depending on the measured variable and the device used [52]. However, the validation of several such devices, including those from Garmin’s range, has been conducted in various sports activities, and clinical study results have highlighted those wearable devices are important for improving athlete performance and preventing adverse cardiovascular events [64]. Additionally, it seems that at least distance measurement—one of the data directions we also adopted—appears to be the most reliable [65]. The use of heart rate belts might provide more accurate data for HR measurement, but the device we used, the Garmin Fenix 6S PRO, offers additional data that a chest belt cannot directly provide, such as: running route, altitude and terrain variation monitoring, training time and environmental conditions, pace and running speed monitoring, recovery monitoring function, etc. These features were useful to us in monitoring (adjusting) the training process.

In this context of limitations, as a subjective evaluation of intensity, we could have also used the rate of perceived exertion (RPE) as an alternative mechanism for controlling effort pace. We believe that this method could have provided valuable information, especially in situations where GPS technology or heart rate monitoring might have been limited by external interference or individual variability.

Another limitation is that the runners participating in validating PASm-12 are not solely road runners but also engage in mountain running. The five tests conducted in PASm-12 were road runs, so the plan should be viewed as an indicative strategy for half marathon preparation and execution. On the other hand, we believe that explaining the performances of the six athletes in our group is not only attributed to the use of PASm-12 or the variety of training methods and tools it includes, but also to other factors that are difficult to control in a study with a single group. Among these, we mention a healthy lifestyle, manifested through appropriate nutritional strategies, suitable professional equipment, and academic education, all of which are often enhanced by domain-specific readings or the frequency of distances covered in hiking as alternative, complementary training. These aspects, interacting variably in the runners’ daily routines, could be subjects of future studies. Last but not least, while PASm-12 was designed for women, it could also be adapted for use with other categories, including men and non-binary individuals.

Additionally, some experts might consider that establishing and following a common training plan, even if only indicative, with fixed indicators of physical preparation such as volume and intensity of effort, would not only be difficult but also inefficient, as each athlete is unique in their own way.

## 5. Conclusions

The implementation of the annual physical training plan for master athletes aged 45+ (PASm-12), created with the hope of becoming a model of good practice for amateur half marathon runners who do not have the guidance of a specialist, has proven to be an effective action. Although the volume and intensity of effort were not fully achieved compared to what was proposed in PASm-12, the data from the *t*-test and the effect size analysis indicated a substantial practical impact of the observed differences, demonstrating that the program successfully achieved results that are both statistically and practically significant.

From a methodological perspective, these indicators were progressively and judiciously scheduled, avoiding a chaotic self-training process. This facilitated the athletes’ biological adaptation, reduced (practically eliminated) the risk of injury, and resulted in the maintenance or improvement of athletic performances, including competitive ones.

PASm-12 also demonstrated its ability to maintain or improve performances in real competitive conditions, even though the results in competitions, such as the Grosseto test, were influenced by external factors specific to the competitive environment, which is often difficult to replicate in non-competitive testing conditions. The success of PASm-12 underscores the complexity and comprehensiveness of this specialized sports training model, structured over a 12-month period, divided into distinct periods correlated with a competition calendar. This methodological approach provides an additional training framework compared to the short-term programming models cited in the referenced studies.

For future applications of PASm-12, it would be beneficial to include additional monitoring tools, such as more advanced wearable technologies and real-time feedback mechanisms, which could further optimize individual biological adaptations. This would allow for more personalized training adjustments, ensuring that each athlete’s performance level is both challenging and achievable. Additionally, exploring the inclusion of motivational and psychosocial support within the program could enhance adherence and long-term success in this type of sports training.

By continuing to refine and adapt PASm-12, this program has the potential to become a benchmark for training master athletes, not only in the self-coaching of half marathon runners but also in various other endurance sports. It offers a solid framework for improving athletic performance while also maintaining the health and well-being of the athletes.

Additionally, researchers and practitioners who wish to replicate or test the PASm-12 model in the context of their own studies or training can receive access to all necessary supplementary information by contacting any of the authors of this study.

## Figures and Tables

**Table 1:** Socio-demographic characteristics of the runners who followed PASm-12 (October 2021).

Athlete	Age (Years)	Residence	Sports Club	Running Experience
S1	50	Brașov	Brasov University Sports Club	13
S2	44	Bucharest	Bucharest Railway Sports Club	10
S3	48	Mediaș	Star Athletics Club Mediaș	30
S4	55	Bucharest	Locomotiva Sports Club Bucharest	12
S5	57	Bistrița Năsăud	Municipal Sports Club Bistrița	38
S6	47	Sibiu	Sibiu Community Club	10

**Table 2:** Common competition calendar for the annual physical training program for the half marathon (PASm-12) 2021–2022.

No.	Month	Competition Name—Field Test	Test Type	Location
1	January	Road running—10 km	Non-specific test	Zărnești (Romania)
2	February	Road running—21 km	Specific test	Cluj-Napoca (Romania)
3	March	Road running—21 km	Specific test	Oradea (Romania)
4	April	Road running—21 km	Specific test	Brașov (Romania)
5	May	European Masters Athletics Championships Non-Stadia—21 km	Specific test	Grosseto (Italy)

**Table 3:** Quantitative macro-indicators of PASm-12.

No.	Indicator	Model—Women +45
1	Training days (no.)	235
2	Training sessions (no.)	235
3	Macrocycles (no.)	1
4	Mesocycles (no.)	12
5	Microcycles (no.)	52
6	Field tests—allocated to the study (no.) *	5
7	Laboratory test (no.)	1

* *In addition to the tests scheduled in PASm-12, following the official test in Grosseto (specific test 5), each athlete also underwent additional unaccounted tests through participation in various competitions—this option for participating in the verification process was driven by the athletes’ familial, professional, and financial constraints.*

**Table 4:** Training means and effort indicators proposed for PASm-12.

Training Means	Mesocycles/Quantitative Effort Indicators												Total Proposed Volume (km)
	I	II	III	IV	V	VI	VII	VIII	IX	X	XI	XII	
Long-duration run with pre-set tempo (5′10″–4′50″/km)		40	50	30		10	10		10				150
Run with elevation difference (km)			60	56	11		10		10				147
Interval run (km)				24	32	18.6	37.8	6.6	24.2	47.8	55.2		246.2
Long-duration run with pre-set tempo (5′40″–5′10″/km)					25				52	18	20	5	120
Long-duration run with pre-set tempo (5′40″–5′30″/km)		40	70	40	68	115	116	16	90	160	187	11	913
Moderate tempo long-duration run (km)	160	48	50	24	43	69	24	41	88	68	72	13	700
Fartlek run (km)		40			31	13	25	10	38	52	24		233
Test run (km)				10	21	21	21	21					94
Total proposed running volume (km/mesocycle)	160	168	230	184	231	246.6	244	94.6	312.2	346	358.2	29	2603.2
Proposed intensity averages (% MHR)	70.0	75.0	70.0	76.3	77.6	72.8	80.8	71.0	76.2	80.5	80.5	78.3	-

**Table 7:** Training zones and maximum heart rate determined through the Bruce test for female runners S1–S6.

Athlete	Training Zone	Heart Rate (bpm)	Tempo/Speed (min/km)
S1	RL/LDL	125	slower than 6:32
	MDL	126–144	6:32–5:30
	TDL	145–155	5:30–5:01
	ETL	156–162	5:01–4:41
	ITL	163–168	4:41–4:24
	SB	**169–max.**	faster than 4:24
S2	RL/LDL	162	slower than 5:33
	MDL	163–169	5:33–4:55
	TDL	170–175	4:55–4:24
	ETL	176–178	4:24–4:06
	ITL	179–181	4:06–3:58
	SB	**182–max.**	faster than 3:58
S3	RL/LDL	134	slower than 5:40
	MDL	135–148	5:40–4:48
	TDL	149–158	4:48–4:20
	ETL	159–166	4:20–4:01
	ITL	167–173	4:01–3:39
	SB	**174–max.**	faster than 3:39
S4	RL/LDL	90	slower than 9:39
	MDL	91–115	9:39–7:03
	TDL	116–126	7:03–6:30
	ETL	127–145	6:30–5:07
	ITL	146–151	5:07–4:48
	SB	**152–max.**	faster than 4:48
S5	RL/LDL	147	slower than 7:19
	MDL	148–153	7:19–6:31
	TDL	154–164	6:31–5:14
	ETL	165–167	5:14–4:52
	ITL	168–170	4:52–4:39
	SB	**171–max.**	faster than 4:39
S6	RL/LDL	133	slower than 6:20
	MDL	134–144	6:20–5:36
	TDL	145–154	5:36–5:13
	ETL	155–168	5:13–4:20
	ITL	169–174	4:20–4:04
	SB	**175–max.**	faster than 4:04

RL/LDL: Low-intensity distance (low-intensity running, recovery pace); MDL: medium-intensity distance (medium-intensity running, base pace); TDL: threshold distance (threshold running, anaerobic threshold pace); ETL: endurance tempo (endurance pace, tempo running); ITL: interval training (interval training, high-intensity pace); SB: speed burst (speed bursts, sprints). The bold value represents the maximum heart rate for each athlete. These values serve as benchmarks for calculating individual percentage values in the case of the scheduled effort intensity.

**Table 8:** Effort intensity realized by runners and proposed by PASm-12.

Mesocycles	Effort Intensity (Average %) Realized/ Athlete (S)						Realized Training Intensity Average S1–S6/Proposed (%)	
	S1	S2	S3	S4	S5	S6	Realized	Proposed
I	65%	75%	75%	60%	67%	70%	69%	70.0%
II	66%	76%	77%	62%	67%	70%	70%	75.0%
III	65%	67%	68%	63%	71%	72%	68%	70.0%
IV	69%	70%	70%	65%	70%	71%	69%	76.3%
V	68%	78%	74%	70%	72%	77%	73%	77.6%
VI	75%	65%	65%	70%	67%	70%	69%	72.8%
VII	75%	78%	75%	80%	76%	70%	76%	80.8%
VIII	75%	65%	65%	70%	67%	70%	69%	71.0%
IX	80%	75%	70%	76%	71%	70%	74%	76.2%
X	77%	72%	77%	81%	79%	75%	77%	80.5%
XI	79%	75%	72%	80%	78%	72%	76%	80.5%
XII	75%	66%	65%	70%	78%	70%	71%	78.3%

**Table 9:** Differences between realized and proposed training intensity through PASm-12.

Pair	Effort Intensity Variable Compared/Mesocycle/Realized-Proposed	Mean S1–S6 (%)	Difference	t	*p*
1	Realized training intensity mesocycle I	69	−1.0	−1.283	0.128
	Proposed training intensity mesocycle I	70.0			
2	Realized training intensity mesocycle II	70	−5.0	−0.867	0.213
	Proposed training intensity mesocycle II	75.0			
3	Realized training intensity mesocycle III	68	−2.0	−2.904	0.017
	Proposed training intensity mesocycle III	70.0			
4	Realized training intensity mesocycle IV	69	−7.3	−2.961	0.016
	Proposed training intensity mesocycle IV	76.3			
5	Realized training intensity mesocycle V	73	−4.6	0.885	0.208
	Proposed training intensity mesocycle V	77.6			
6	Realized training intensity mesocycle VI	69	−3.8	−1.972	0.053
	Proposed training intensity mesocycle VI	72.8			
7	Realized training intensity mesocycle VII	76	−4.8	2.833	0.018
	Proposed training intensity mesocycle VII	80.8			
8	Realized training intensity mesocycle VIII	69	−2.0	−1.972	0.053
	Proposed training intensity mesocycle VIII	71.0			
9	Realized training intensity mesocycle IX	74	−2.2	1.164	0.148
	Proposed training intensity mesocycle IX	76.2			
10	Realized training intensity mesocycle X	77	−3.5	3.984	0.005
	Proposed training intensity mesocycle X	80.5			
11	Realized training intensity mesocycle XI	76	−4.5	2.956	0.016
	Proposed training intensity mesocycle XI	80.5			
12	Realized training intensity mesocycle XII	71	−7.3	−0.526	0.311
	Proposed training intensity mesocycle XII	78.3			

**Table 10:** Differences between the average realized test times by runners S1–S6 and the proposed average times in PASm-12 (H3.b).

Test No.	Sports Event/Test km/Location	Proposed PASm-12 Average Time s (I)	Realized Average Time S1–S6 s (J)	Difference I–J	SD	t	*p*
1	Zărnești 10 km test	3000	2951.6	48.40	110.97	0.975	0.39
2	Cluj-Napoca 21 km test	7035	6458	576.20	344.297	3.742	0.02
3	Oradea 21 km	7035	6858	177.00	433.06	0.914	0.41
4	Brașov 21 km	7035	6446.8	588.20	422.96	3.110	0.04
5	Grosseto 21 km	6300	6535.2	−235.20	441.19	−1.192	0.3

Note: N = 6, bootstrapping—1000; execution time averages are described in seconds; SD—standard deviation.

**Table 11:** Number of competitions conducted by female runners S1–S6 during the competition period not included in PASm-12.

Competition Level	Subjects						Total
	S1	S2	S3	S4	S5	S6	
National competitions	0	9	9	3	1	6	28
International competitions	0	0	1	3	1	1	6
Total	0	9	10	6	2	7	34

## Data Availability

The data presented in this study are available upon request from any of the study authors.
